# Persistent adaptation through dual-timescale regulation of ion channel properties

**DOI:** 10.1073/pnas.2530340123

**Published:** 2026-03-02

**Authors:** Yugarshi Mondal, Ronald L. Calabrese, Eve Marder

**Affiliations:** ^a^Volen Center and Biology Department, Brandeis University, Waltham, MA 02454; ^b^Biology Department, Emory University, Atlanta, GA 30322

**Keywords:** intrinsic excitability, activity-dependent regulation, high potassium, homeostatic plasticity, computational model

## Abstract

Neurons are terminally differentiated cells that adapt to maintain stable function over years, despite encountering a wide range of environmental perturbations. In some cases, recovery from perturbation is not shaped by prior exposure; in others, recovery depends on the neuron’s perturbation history. A particularly striking form of history-dependent recovery occurs when prior exposure enhances the neuron’s ability to recover from future perturbations while leaving baseline activity largely unchanged. Among the many mechanisms that may contribute to such history-dependent improvement in recovery, we investigate one based on the regulation of intrinsic currents. Using a model of activity-dependent homeostasis, we show that improved recovery can be encoded through lasting changes in channel density, while rapid shifts in ion channel voltage-dependence provide immediate compensation during perturbations. We refer to the long-lasting intrinsic trace that accompanies this improved recovery a persistent adaptation. Interestingly, these roles are noninterchangeable: when voltage-dependence evolves slowly and maximal conductances change rapidly, an intrinsic trace is not stored, eliminating persistent adaptation even when improved recovery is preserved.

## Significance

Some neurons exhibit a remarkable property: they recover more quickly from repeated challenges even though their baseline activity appears unchanged. How such history-dependent improvement in recovery arises is unclear. Using a computational model, we show that this behavior can reflect a persistent adaptation encoded in slow changes in ion channel expression, which store a lasting trace of past perturbations. Faster shifts in channel voltage-sensitivity then enable rapid recovery upon re-exposure without altering baseline activity.

Over their lifetimes—often spanning years—neurons encounter countless perturbations in their external environments that push them away from their typical activity patterns. Broadly, neurons can recover activity in two ways: they can reestablish a stable activity state anew each time a perturbation occurs, or they can use information acquired during prior perturbations to guide subsequent recovery. A particularly striking form of this latter history-dependent recovery occurs when prior exposure enhances the neuron’s ability to recover from future perturbations while simultaneously leaving baseline activity largely unchanged. This happens in a variety of systems. For example, neurons exposed to repeated high extracellular potassium perturbations recover their bursting rhythm more quickly on subsequent exposures (1, 2), even though their baseline activity appears unchanged. Relatedly, rapid cold hardening (RCH) is observed in some insects. RCH occurs when a brief exposure to mild cold enhances tolerance to subsequent chill stress (3, 4). In African migratory locusts, RCH lowers the temperature threshold that triggers spreading depolarization that accompanies neural shutdown immediately preceding chill induced coma. However, despite this electrophysiological change, they appear behaviorally normal under standard conditions (5).

There may be many mechanisms that may contribute to such history-dependent improvement in recovery; we investigate one based on the regulation of intrinsic currents. In both examples given above, there is indirect evidence that improvement in recovery may involve changes to the neuron’s intrinsic properties. Neurons that resume firing sooner after repeated exposure to high extracellular potassium perturbations exhibit altered responses to current injection (2, 6). RCH is thought to be accompanied by adjustments in neurons’ ability to maintain potassium homeostasis, potentially via changes in active potassium currents, passive potassium currents, or sodium–potassium pump activity, which in turn influence intrinsic excitability (7). However, in both examples, these changes have not yet been linked to specific alterations in the ionic currents that set a neuron’s firing properties, leaving open the question of which biophysical changes support this improvement in recovery.

To investigate this, we used an activity-dependent homeostatic model which modified two key properties of a neuron’s ion channel repertoire: the number (or density) of channels expressed, and the voltage- and time-dependence of channel gating (8). A computational model allows us to track all the intrinsic parameters simultaneously. Experimentally, this is not feasible because each ionic current must be isolated separately, preventing an instantaneous measurement of the neuron’s entire channel repertoire. Moreover, determining these parameters would require repeated use of blockers and voltage-clamp steps that would very likely begin to alter the neuron’s intrinsic configuration—provided the neuron even survived such a protocol.

In this work, we assumed that changes in channel density occur on slower timescales than changes in voltage-dependence, consistent with the general timescale differences between protein synthesis/degradation and posttranslational modifications (8). Theoretical work has implicated both as being important to recovery from perturbation. On longer timescales, neurons may adjust channel densities to reestablish stable activity (9–16). On shorter timescales, neurons can adjust their excitability by regulating their channel voltage-dependence (17–28). These changes can facilitate recovery from perturbations, and in some circumstances modifications of only a single or pair of currents may be sufficient—for example, during recovery from high potassium perturbation. Both mechanisms likely operate in combination to support recovery.

Here we use computational experiments to show that enhanced recovery following prior exposure to a perturbation—modeled here as elevated extracellular potassium—can be explained by the interaction of fast and slow intrinsic processes. Slow changes in channel density encode a latent trace of past activity, forming a persistent adaptation to the perturbation, while rapid shifts in voltage-dependence provide immediate compensation during re-exposure. Importantly, these roles are not interchangeable: reversing the timescales of conductance and voltage-dependence regulation eliminates the latent trace and abolishes persistent adaptation. Together, these processes show how coordinated intrinsic regulation can support improved recovery through persistent adaptation of a neuron’s intrinsic properties.

## Results

To explore how persistent adaptation can emerge from intrinsic plasticity, we used a conductance-based neuron model with activity-dependent regulation of both ion channel density and voltage-dependence (8).

Briefly, the model consists of a single-compartment Hodgkin–Huxley type neuron with seven intrinsic currents: a hyperpolarization-activated inward current, two calcium currents, one sodium current, three potassium currents, and a leak current. Intracellular calcium fluctuates in response to neuronal activity. Three distinct filters are applied, each designed to extract a different component of the calcium signal: one isolates calcium entry associated with spiking, another captures calcium driven by the underlying slow wave, and a third reflects the overall average intracellular calcium concentration. The decomposition of the calcium signal across distinct timescales reflects a strategy that has been used to analyze intrinsic neuronal dynamics in other biophysical contexts (29). Time-averaged values of each filtered signal are compared to predefined setpoints. These setpoints—specified a priori—represent the neuron’s activity targets (8, 10, 30). When neuronal activity causes sustained deviations from these targets, the model’s activity dependent-regulation mechanism adjusts each intrinsic current’s maximal conductance (reflecting ion channel density) and its voltage-dependent activation and, where applicable, inactivation curves, to bring activity back in line with the targets.

Crucially, the activity-dependent regulation mechanism employed here operates on two timescales: a slow process adjusts maximal conductances ($\tau_{g}=600$ s), reflecting protein synthesis and degradation; a fast process shifts the voltage-dependence of activation and inactivation ($\tau_{\mathrm{half}}=6$ s), mimicking posttranslational modifications. All details of the model are provided in Mondal et al. (8), motivated by the experimental work in Rue et al. (1), He et al. (6), and Lee and Marder (2).

In what follows, we take 20 degenerate spontaneously bursting neurons from Mondal et al. (8) and subject them to a sequence of three increases in extracellular potassium concentration.

### Enhanced Recovery Following Prior Perturbation.

**Fig. 1.**
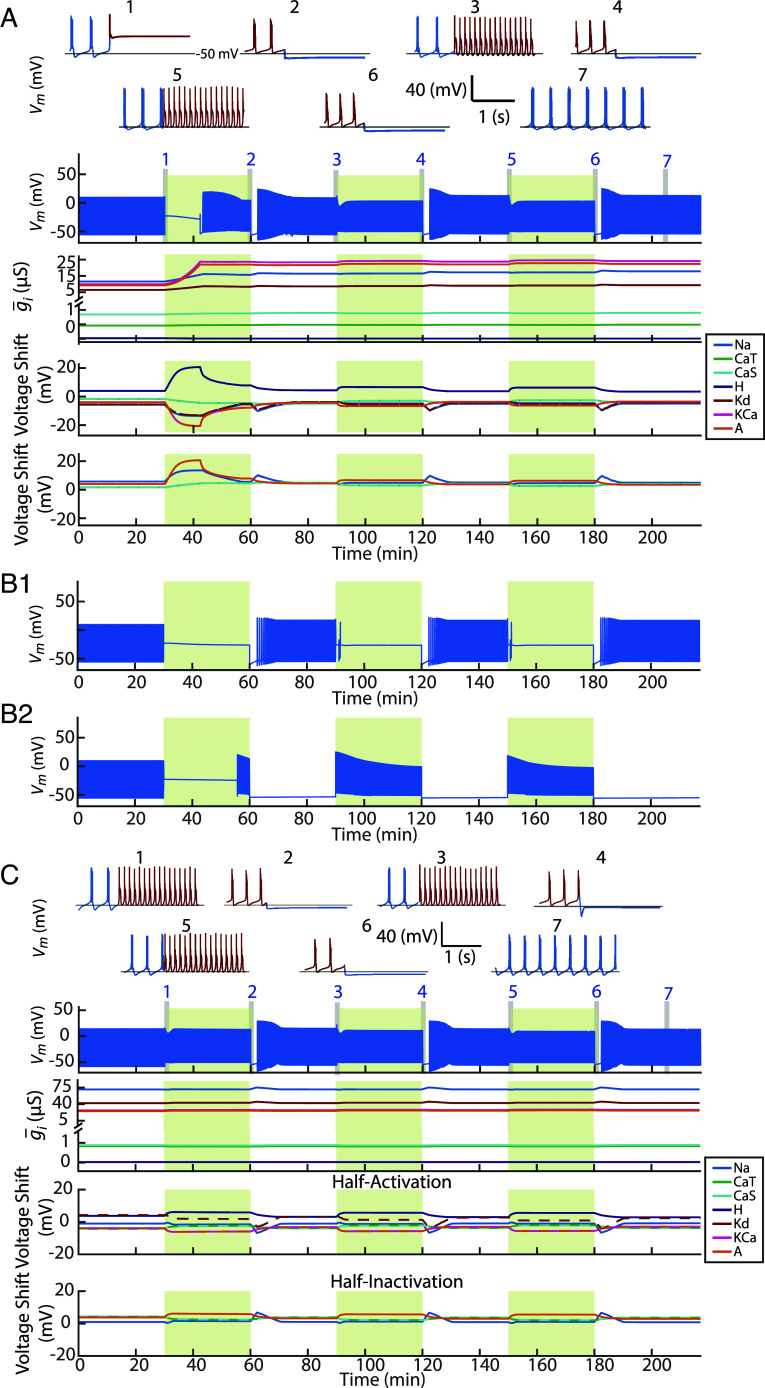
Interaction of slow and fast intrinsic plasticity improves recovery following perturbation. (*A*) An intrinsically bursting neuron model was subjected to three repeated high extracellular potassium perturbations (green shaded regions). Top two rows show expanded voltage traces (*Insets* 1 to 7) at selected time points. Red segments indicate activity during perturbation; blue segments indicate activity during wash. The third row shows full voltage trace over 220 min. The fourth row shows changes in maximal conductances for each intrinsic current. Fifth and sixth rows show shifts in voltage-dependent half-activation and half-inactivation voltages. The baseline half-activation and half-inactivation are given in Table 1. (*B1*) Same model with only voltage-dependence half-(in)activations allowed to vary. (*B2*) Same model with only maximal conductances allowed to change. (*C*) Neuron model with intrinsic parameters preadapted to high-potassium perturbation. The plot labels are described as in *A*. This model exhibited no latency to first burst during the first perturbation.

Fig. 1 illustrates how a model neuron’s ion channel densities and voltage-dependences changed in response to repeated increases in extracellular potassium concentration. Each perturbation elevated extracellular potassium to approximately 2.5× the baseline level and lasted 30 min, followed by a 30-min wash period in baseline conditions (see *Methods* for details).

Before the first perturbation, the neuron fired bursts under baseline conditions (Fig. 1*A*, *Inset* 1, blue). Upon exposure to high potassium, it entered depolarization block (*Inset* 1, red), but recovered bursting during the perturbation (*Inset* 2, red). Remarkably, when the same perturbation was repeated, the neuron resumed bursting almost immediately (*Insets* 3 and 5, red), indicating that prior exposure altered the neuron’s response to subsequent perturbations—constituting a form of enhancement of recovery.

In 18 out of 20 simulations, bursting returned during each wash period (*Insets* 3, 5, 7, blue). Each wash is accompanied by an initial hyperpolarizing undershoot, suggesting it is a consequence of releasing the high potassium state. During the perturbation, currents are rebalanced to support bursting in a depolarized environment; when conditions return to normal, this balance is transiently inverted, producing hyperpolarization before stable bursting resumes. Of the remaining two simulations, one exhibited stable bursting during the first two washes, but during the third wash the bursting envelope evolved slowly over minutes rather than settling into a steady pattern. In the other, stable bursting was regained only after the third wash.

The panels below the voltage trace show that this recovery enhancement coincided with large changes in maximal conductances during the first perturbation, followed by stabilization within a narrower range during later cycles. Intrinsic currents’ half-(in)activations continued to shift across perturbations and washes, reflecting ongoing, fast-timescale tuning of excitability. In this model, both processes were required to reproduce this recovery enhancement. When only half-(in)activations were regulated (Fig. 1*B1*), the neuron failed to recover during perturbation and resumed bursting only during wash. When only maximal conductances were regulated (Fig. 1*B2*), the neuron recovered bursting during the first perturbation and exhibited immediate bursting on subsequent ones—suggesting a form of recovery enhancement—but lost bursting stability during wash, instead falling silent. Together, these results suggest that, for this model, fast and slow feedback mechanisms play complementary roles using prior perturbation to enhance recovery: each alone supports partial recovery, but their interaction enables robust restoration and long-term stability of target activity.

One model in the set of 20 (Fig. 1*C*) showed no latency to first burst during the first perturbation, suggesting that this model possessed an ion channel configuration already tolerant to the high potassium perturbations unlike the other models that reached this state only after their first exposure.

**Fig. 2.**
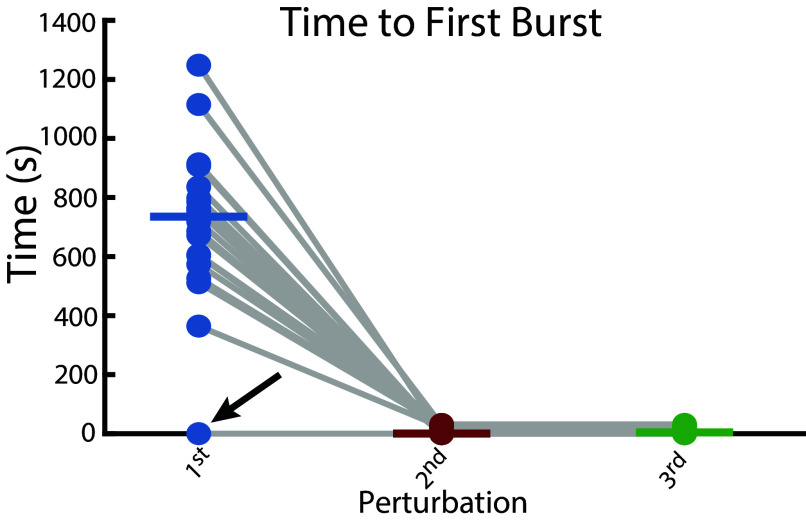
Exposure to prior perturbation improves time to recovery. Each dot represents time to first burst during each of three repeated high-potassium perturbations across 20 model neurons. The horizontal bar in each column represents the median of each group. Each gray line tracks a single model across perturbations. The arrow marks the model that was already adapted to the perturbations (Fig. 1*C*).

Fig. 2 shows that the pattern of recovery enhancement observed in Fig. 1*A* was common to most models. In 19 out of 20 neurons, the time to first burst during the first perturbation was significantly longer than during subsequent perturbations. The remaining neuron (Fig. 2, marked with arrow) exhibited near-immediate bursting even on the first exposure, suggesting it was already well-adapted to the perturbation and that this persisted on subsequent perturbations (seen in Fig. 1*C*). In most cases, changes were made during the initial perturbation that enabled faster recovery on subsequent trials.

### First Perturbation Triggers Large Maximal Conductance Shifts That Then Stabilize.

**Fig. 3.**
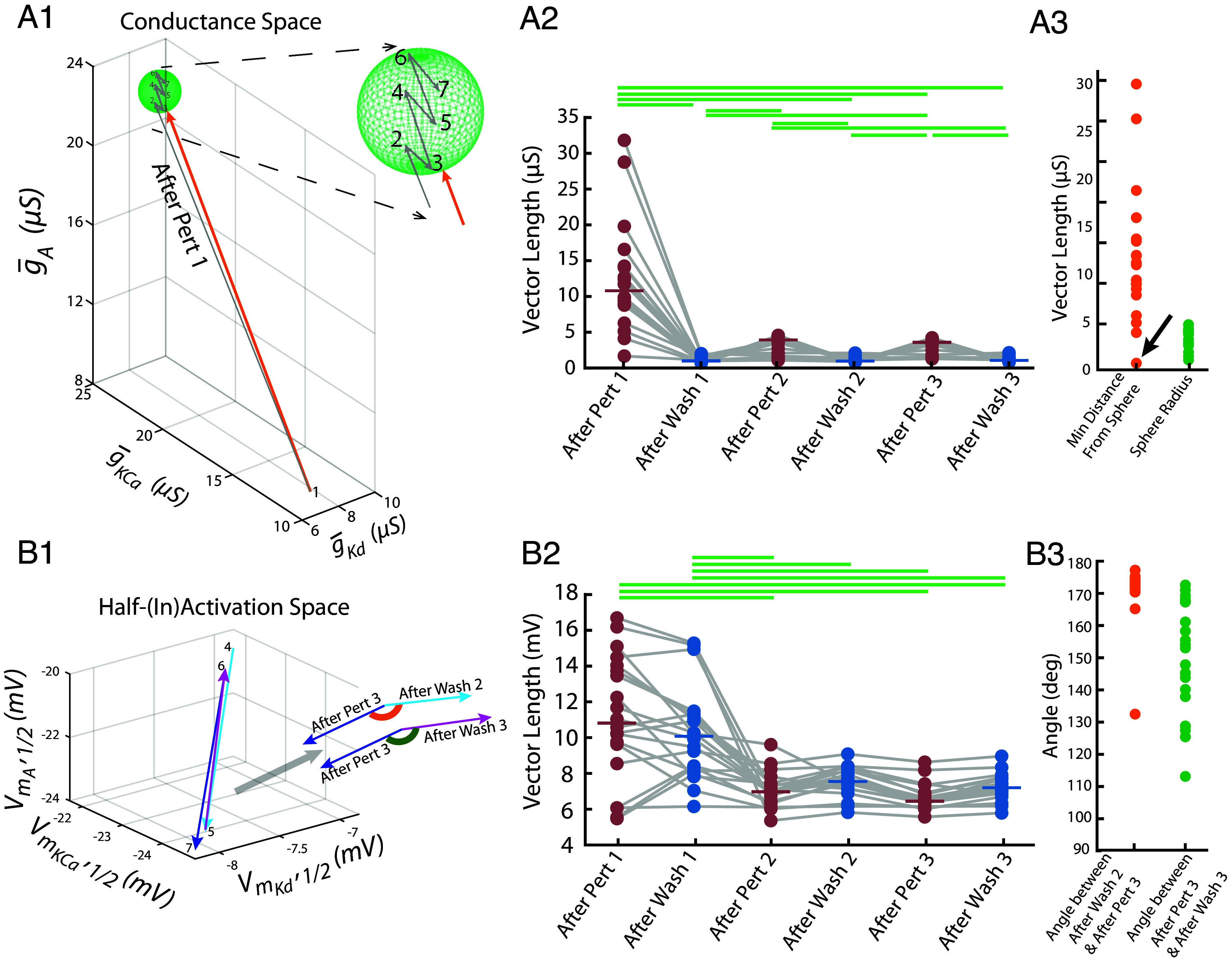
Improved recovery following perturbation is accompanied by a large initial conductance shift followed by switching of voltage-dependent properties across repeated perturbations. (*A1*) Three-dimensional projection of the conductance trajectory for the model neuron shown in Fig. 1*A*. Each numbered point is the neuron’s conductance state projected into a 3D subspace at the corresponding time in the perturbation–wash sequence. The green sphere shows the projection of the minimum bounding sphere enclosing points 2 to 7 (the adapted region). The orange vector is the minimum-distance path from the initial state (point 1) to the surface of this sphere. Gray vectors show the projected trajectory segments, the first of which corresponds to After Pert 1; the remaining unlabeled segments correspond to the other displacement vectors whose magnitudes are plotted in *A2*. (*A2*) Vector lengths for all 20 models showing the magnitude of change in maximal conductances (μS) across successive perturbation and wash cycles. Horizontal green bars indicate statistically significant pairwise differences between medians measured using Kruskal–Wallis followed by Dunn’s test with Bonferroni correction (adjusted *P* < 0.05). (*A3*) Summary measurements in conductance space for each model. Green points show the radius of the final conductance bounding sphere (defined in *A1*), which encloses the conductance states following the first perturbation (points 2 to 7). Orange points indicate the minimum distance from the initial conductance state (point 1) to the outside of this sphere. The arrow marks the model that was already adapted to the perturbations (Fig. 2). (*B1*) Three-dimensional projection of half-(in)activation trajectories for the model neuron shown in Fig. 1*A*. Each numbered point corresponds to a three-dimensional projection of the neuron’s half-(in)activation parameters at a specific time point during the perturbation and wash cycles. The final four time points are shown. The light blue, magenta, and dark blue vectors span the distances between points 4 and 5, 5 and 6, 6 and 7, respectively. These vectors denote successive displacement vectors in half-(in)activation space that are used for the angle analysis: After Wash 2, After Perturbation 3, and After Wash 3, respectively. To compute angles, the relevant pairs of displacement vectors were aligned tail-to-tail in the full high-dimensional parameter space. The angle associated with the wash–perturbation–wash sequence (After Perturbation 3 to After Wash 3) is shown in green, and the angle associated with the perturbation–wash–perturbation sequence (After Wash 2 to After Perturbation 3) is shown in orange. (*B2*) Vector lengths for all 20 models showing the magnitude of change in half-(in)activations across successive perturbation and wash cycles. Statistical comparisons were performed using Kruskal–Wallis followed by Dunn’s test with Bonferroni correction; horizontal bars denote significant pairwise differences (adjusted *P* < 0.05). (*B3*) Angles, as defined in (*B1*), for all 20 model neurons. Each point represents the measured angle for one model in either the perturbation–wash–perturbation sequence (orange) or the wash–perturbation–wash sequence (green).

In Fig. 1*A*, we saw that the maximal conductances changed during the first perturbation and stabilized during subsequent perturbation and wash cycles. This, in fact, was representative of most models’ behavior. To quantify this pattern, we examined the trajectory of each neuron’s conductance vector across seven key time points: just before the first perturbation, at the end of the first perturbation, just before the second perturbation, at the end of the second perturbation, and so on—capturing its intrinsic state throughout all perturbation and wash phases up to the end of the third perturbation. We then computed six displacement vectors by subtracting each point from the one that immediately follows it—i.e., computing “next” minus “initial.” Each resulting vector quantifies how much—and in what direction—the conductance configuration changed during that time interval. Fig. 3*A1* shows a three-dimensional projection of this trajectory for the model neuron in Fig. 1*A*.

In Fig. 3*A2*, these vectors are labeled on the x-axis. For example, “After Pert 1” reflects the change in conductances from just before the start of the first perturbation to just before it ended—capturing the change during the first high-potassium exposure. “After Wash 1” reflects the change from the end of the first perturbation to just before the second perturbation, and so on through the third perturbation and its wash. In total, these six vector lengths summarize how much the neuron’s conductance profile shifted during each perturbation and wash phase. As shown in Fig. 3*A2*, the largest changes occurred during the first perturbation, followed by relatively minor adjustments in subsequent phases.

To simplify the patterns observed in Fig. 3*A2*, we constructed a minimum bounding sphere around the final six conductance states (points 2 to 7). The green sphere in Fig. 3*A1* shows the three-dimensional projection of this region. This sphere defined the region of conductance space occupied by the neuron once it had adapted. We then measured the minimum distance from the initial state (point 1) to the surface of this sphere. The three-dimensional projection of this path is shown as the orange vector in Fig. 3*A1*.

The green sphere radii and orange distances for all models are plotted in Fig. 3*A3*. If this distance exceeded zero, it indicated that the first state lay outside the region occupied in later perturbation-wash cycles (*Methods*). This was true for all models tested, supporting the idea that a major shift in conductance configuration occurred during the first perturbation, followed by stabilization within a narrower regime (Fig. 3*A3*). In 16 out of 20 models, the distance from the preperturbation-1 conductance state to the center of the adapted region exceeded the radius of the bounding sphere. This means that for most neuron models, the neuron’s initial conductance profile lay not just outside the range of its later configurations, but well beyond it. In these cases, this distance was greater than at least twice the radius—indicating that the neuron began far from the set of states it would go on to occupy.

Of the remaining four models, one corresponded to the preadapted case discussed earlier (Fig. 1*C*). As noted, firing in this model recovered almost immediately after the first perturbation. Its initial conductance state lay just outside the adapted region (Fig. 3*A3*, arrow), unlike the other models that began far away. Unlike the others, only a small adjustment was required—underscoring how proximity to the adapted regime can sharply reduce the scale of change needed for recovery.

### Faster Recovery on Subsequent Trials Involves Revisiting Half-(In)Activation Profiles.

As seen in Fig. 1*A*, the half-(in)activation parameters did not converge to a single configuration but instead alternated between distinct states during perturbation and wash phases. These trajectories suggest a switching behavior, with the neuron moving toward one half-(in)activation configuration during perturbation and returning to another during wash. To quantify this switching, we analyzed trajectories through half-(in)activation space. Fig. 3*B1* shows a three-dimensional projection of these trajectories for the model neuron in Fig. 1*A*. In this projection, the final transitions between regimes suggest that the neuron exited each regime along a trajectory that approximately reversed its entry path.

Because this visualization reflects a low-dimensional projection of a higher-dimensional space and a single model instance, we next tested this observation quantitatively across all models in the full half-(in)activation space. For wash, we compared the transition from Wash 2 to Perturbation 3 with the subsequent transition from Perturbation 3 to Wash 3. These transitions—represented by the vectors labeled After Perturbation 3 and After Wash 3 in Fig. 3*B2*—capture the final excursion out of and back into the wash condition. The angle between these vectors was computed by aligning them tail-to-tail in high-dimensional space (Fig. 3*B1*). Similarly, for perturbation, we compared the transition from Perturbation 2 to Wash 2 with the following transition from Wash 2 to Perturbation 3—represented by the vectors After Wash 2 and After Perturbation 3—and again measured the angle between them.

In all models, these angles exceeded 90 degrees, indicating that the system reversed direction between successive visits to the same regime (Fig. 3*B3*). When combined with the observation that vector magnitudes remained stable (Fig. 3*B2*), this suggests that neurons alternated between two half-(in)activation configurations—one for wash, one for perturbation—rather than converging on a single set of parameters.

Taken together, Fig. 3 suggests that, following initial change in channel expression, the neurons settled into a stable channel repertoire, while half-(in)activations shifted between two configurations to maintain bursting across both wash and perturbation conditions. Fig. 1*A* shows this happening in a specific neuron model.

### Maximal Conductances Store a Latent Trace of Prior Perturbation.

**Fig. 4.**
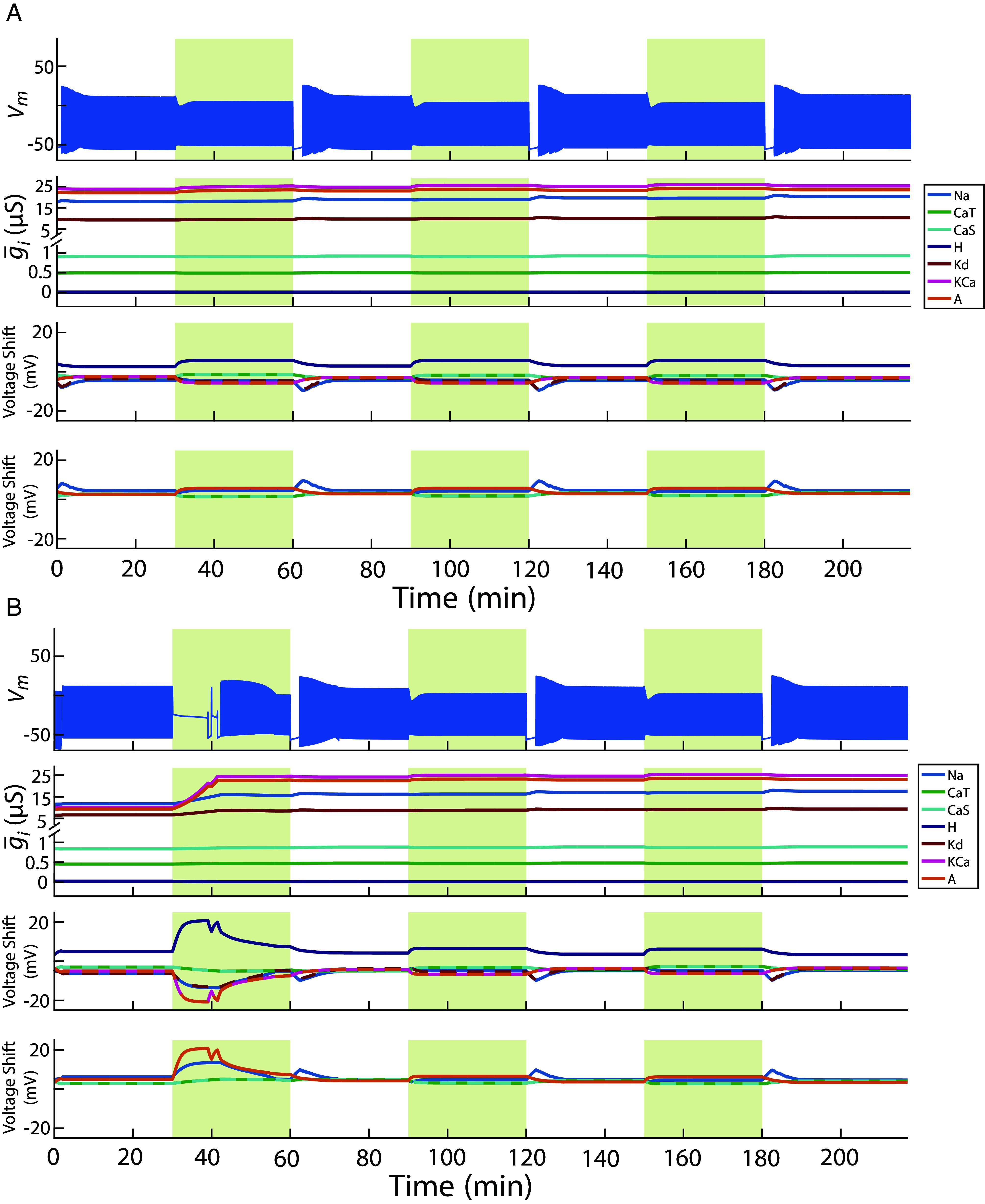
Do channel densities or channel voltage-dependences store a trace of prior perturbations? (*A*) The model from Fig. 1*A* was first reconfigured to retain the final maximal conductances from the end of the original simulation while resetting all half-(in)activation voltages to their preperturbation values. It was then re-exposed to the same perturbation sequence as in Fig. 1*A*. Rows are arranged as in Fig. 1*A*. (*B*) The same model was reconfigured to preserve the final half-(in)activation voltages while restoring maximal conductances to their original preperturbation values, and then re-exposed to the same perturbation sequence. Rows are arranged as in Fig. 1*A*.

The foregoing results suggest that the neuron improves recovery following the first perturbation because the initial perturbation alters the neuron’s expression of ion channels. To test whether this is true, we re-exposed each model to the same sequence of perturbations, starting from two distinct sets of initial conditions. In the first condition (Fig. 4*A*), we preserved the final maximal conductances from the original simulation but reset all half-(in)activation voltages to their original, preperturbation values. In the second condition (Fig. 4*B*), we did the opposite: we kept the final half-(in)activation voltages but restored the original maximal conductances. In both cases, the homeostatic mechanism remained active throughout. Independently resetting one parameter set while carrying over the other from the end of the original simulation allowed us to isolate the contributions of maximal conductances versus half-(in)activation voltages in supporting improved recovery to perturbations.

The results revealed a striking asymmetry. When the final maximal conductances were retained but the half-(in)activations were reset (Fig. 4*A*), the model rapidly reestablished bursting during the first perturbation—suggesting that the conductance profile alone was sufficient to support improved recovery to perturbations. In contrast, when the final half-(in)activation voltages were retained but the maximal conductances were reset (Fig. 4*B*), the model exhibited depolarization block during the first perturbation and required time to recover bursting —resembling the behavior seen in Fig. 1*A* during initial exposure. This suggested conductance profile shifts were also perhaps a necessary condition for improved recovery.

**Fig. 5.**
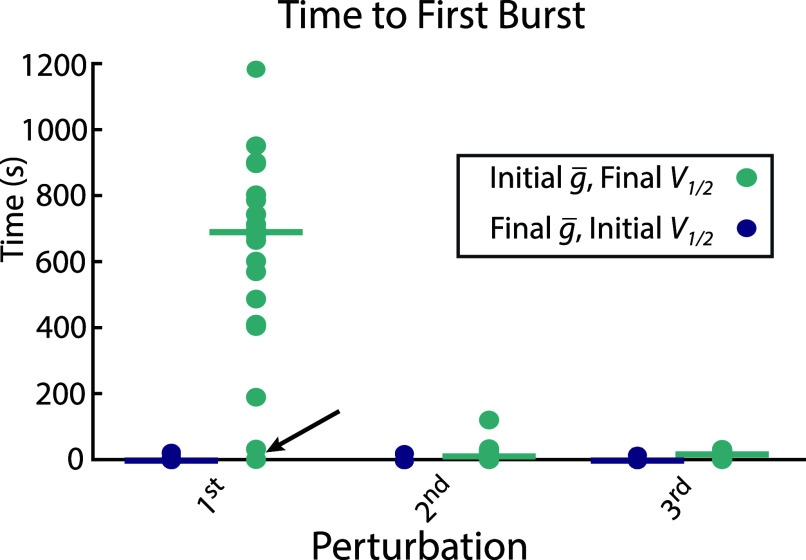
Time to recovery across repeated perturbations when maximal conductances and half-(in)activations are reconfigured. This analysis replicates Fig. 2 for the same 20 model neurons, but with parameter rearrangements described in Fig. 4. Models were reconfigured either to retain final maximal conductances while resetting half-(in)activation voltages to their preperturbation values (purple), or to retain final half-(in)activation voltages while resetting maximal conductances (cyan) and were then re-exposed to the same sequence of perturbations. The arrow marks the models that were already adapted to the perturbations.

For all tested models, those initialized with the final maximal conductances (Fig. 5, purple) resumed bursting almost immediately during the first perturbation, as in Fig. 4*A*. This indicates that the conductance profile alone was sufficient to support immediate improved recovery. In contrast, 18 of the 20 models initialized with the final half-(in)activation voltages but restored to their original, preperturbation maximal conductances (Fig. 5, cyan) required substantially more time to recover bursting—mirroring the slow response seen during the first perturbation in the original simulations (Fig. 4*B*). This pattern suggests that, in most cases, slow changes in maximal conductances were also necessary for rapid recovery. Of the two exceptions (Fig. 4*A*, arrow), one was the preadapted case (Fig. 1*C*), where the original conductances were already tuned for the perturbation. The remaining recovered rapidly despite the conductance reset, implying that in some cases the original conductance repertoire can still support rapid recovery if paired with a different half-(in)activation configuration.

**Fig. 6.**
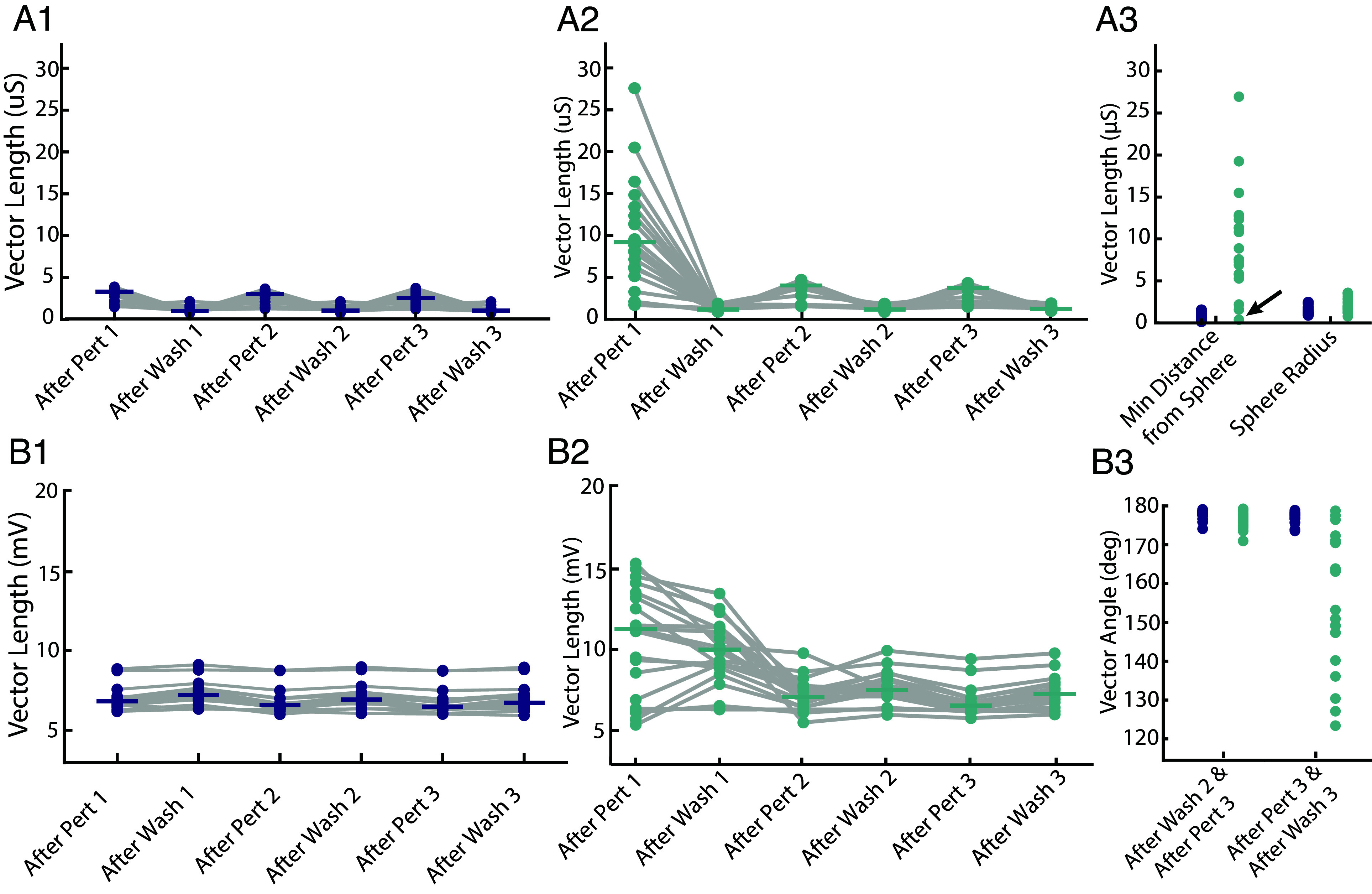
Trajectories in parameter space across multiple perturbations with reconfigured models. The neuron models were reconfigured in the following ways: either initialized with final maximal conductances and preperturbation half-(in)activation voltages (purple) or initialized with preperturbation maximal conductances and final half-(in)activation voltages (cyan). The trajectories of the reconfigured models through parameter space are summarized here. During re-exposure to the same sequence of high-potassium perturbations. (*A1*) Vector lengths in maximal conductance space after each perturbation and wash phase for models initialized with final maximal conductances and preperturbation half-(in)activation voltages (same analysis as in Fig. 3*A2*). (*A2*) Vector lengths in maximal conductance space after each perturbation and wash phase for models initialized with final half-(in)activation voltages and preperturbation maximal conductances (same analysis as in Fig. 3*A2*). (*A3*) A comparison of reconfigured models’ vector distances in maximal conductance space at the start of re-exposure, showing each model’s minimum distance to the bounding sphere defined by postadaptation states (*Left*) and the radius of that sphere (*Right*) (same analysis as in Fig. 3*A3*). The arrow mark models whose preperturbation states lay close to the postadaptation sphere. (*B1*) Vector lengths in half-(in)activation voltage space after each perturbation and wash phase for models initialized with final maximal conductances and preperturbation half-(in)activation voltages (same analysis as in Fig. 3*B2*). (*B2*) Vector lengths in half-(in)activation voltage space after each perturbation and wash phase for models initialized with final half-(in)activation voltages and preperturbation maximal conductances (same analysis as in Fig. 3*B2*). (*B3*) A comparison of reconfigured models’ angles between displacement vectors of half-(in)activation parameters during transitions between perturbation and wash phases (same analysis as in Fig. 3*B3*).

To further interpret the differences in recovery times, we revisited the geometric framework shown as a three-dimensional projection in Fig. 3*A1* to analyze how maximal conductance configurations evolved during re-exposure. Using the same approach to define each model’s stable regime in conductance space, we constructed a bounding sphere around the conductance states observed after the initial perturbation and measured how close the conductance state at the beginning of re-exposure lay to this region. As shown in Fig. 6*A3*, 20 of 20 models initialized with their final maximal conductances (purple) began near the trajectory of subsequent states during re-exposure. In contrast, 17 of 20 models reset to their original preadaptation maximal conductances (cyan) often started far outside this region and had to traverse large distances in conductance space to converge toward it. The remaining three models (Fig. 6*A3*, arrow) began re-exposure already close to their adapted state. Two of these corresponded to the rapidly recovering models described above. In the third case, recovery was faster after prior exposure, but the maximal conductances did not stabilize after the first perturbation. Instead, they continued to change during subsequent perturbations. Nevertheless, the initial shift was sufficient to improve recovery upon re-exposure.

We also examined the dynamics of half-(in)activation voltages during re-exposure by measuring the angles between successive displacement vectors. For each model and initialization, the lengths of the displacement vectors during the final transitions into and out of the wash and perturbation phases were consistent, allowing us to focus on direction rather than magnitude, as in Fig. 3*B1* (Fig. 6 *B1* and *B2*). Across both reinitialization conditions, these transitions showed directional reversals in half-(in)activation parameter space—though to varying extents. Models initialized with their final maximal conductances (Fig. 6*B3*, purple) exhibited angles tightly clustered near 180 degrees, indicating consistent reversals in trajectory: the neuron departed from and returned to similar half-(in)activation configurations, whether starting from wash or perturbation phase. In contrast, models reset to their original conductances (Fig. 6*B3*, cyan) showed greater variability—particularly during transitions into and out of the final wash. This increased dispersion might have been expected because the models use the same initial maximal conductances as in the original runs where models were still establishing a stable switching strategy (Fig. 3*B3*). Together, these results suggest that while half-(in)activation parameters enable flexible modulation of excitability across environmental contexts, there is a persistent adaptation that is encoded in the maximal conductance profile.

### Reversing the Timescales Disrupts Persistent Adaptation.

The results above show that when prior exposure to perturbation enhances a neuron’s ability to recover from subsequent perturbations—while leaving baseline activity largely unchanged. Furthermore, this is associated with a persistent adaptation encoded in slowly evolving maximal conductances, with voltage-dependence adjusting rapidly to support recovery. This raises a key question: is persistent adaptation simply a consequence of having both fast and slow intrinsic mechanisms, or does it depend on which mechanisms operate on which timescales? To address this, we reversed the timescales of regulation, allowing half-(in)activation shifts to evolve slowly while maximal conductances changed rapidly, and applied the same perturbation sequence to the same set of 20 models.

**Fig. 7.**
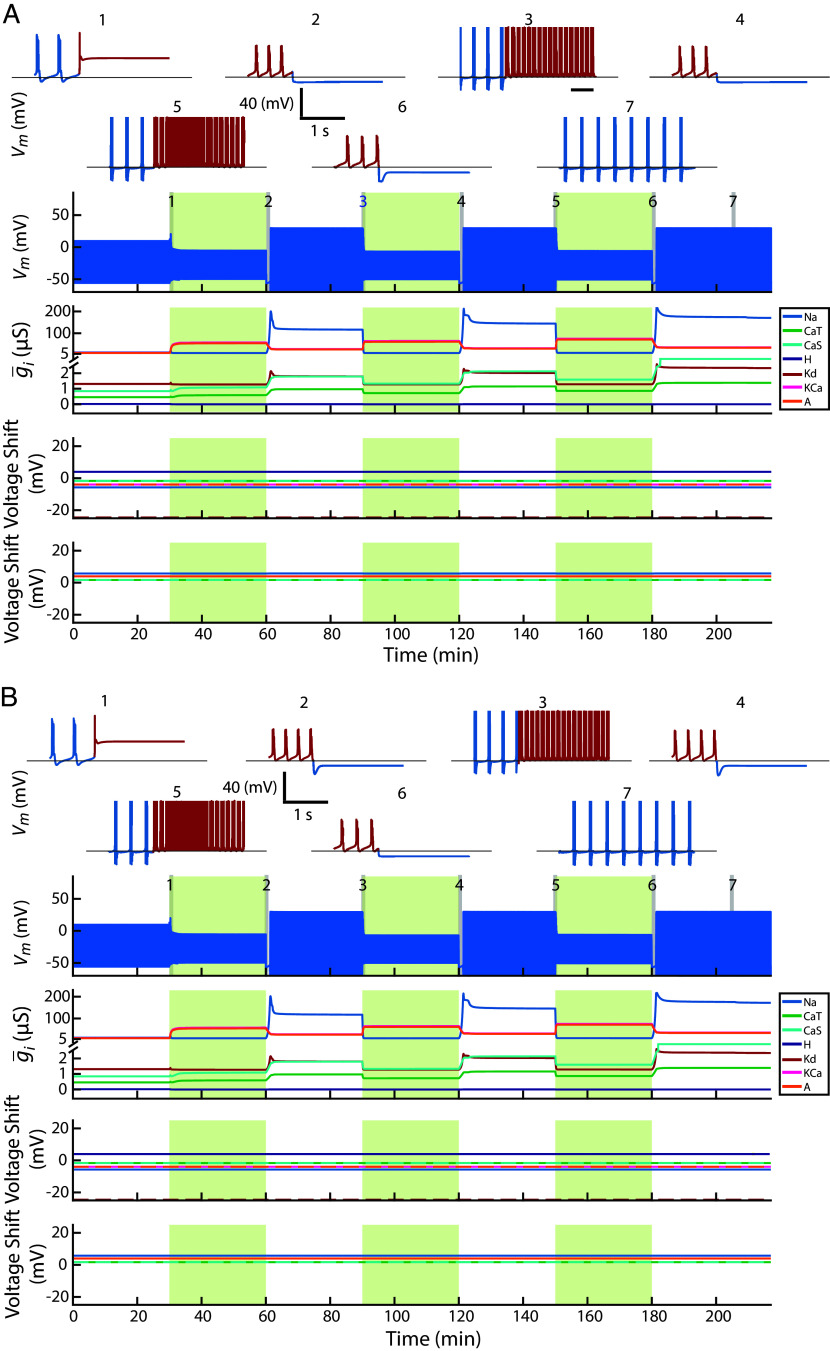
Assigning half-(in)activations the slow variable abolishes trace of prior perturbation. The model neuron from Fig. 1*A* was re-exposed to the same perturbation–wash sequence, starting from the same initial conditions. Rows are arranged identically to Fig. 1*A*. (*A*) Simulation with slow half-(in)activation shifts ($\tau_{\mathrm{half}}=600$ s) and fast maximal conductance changes ($\tau_{g}=6$ s). This simulation exhibits improved recovery on subsequent perturbations as well. However, the model does not appear to store a trace of this adaptation in the slow variable, the half-(in)activations. (*B*) Same simulation as in (*A*), except half-(in)activation shifts were disabled entirely ($\tau_{\mathrm{half}}=\infty$ s). The resulting activity is nearly indistinguishable from (*A*), indicating that slow half-(in)activation indeed does not store a trace of prior perturbations.

If the two mechanisms were interchangeable, slow half-(in)activation shifts would be expected to store a persistent trace of the initial perturbation. This did not occur (Fig. 7). In particular, when the half-(in)activation shifts evolved slowly, half-(in)activation parameters did not change in response to perturbation indicating they did not store a detectable trace of prior perturbations (Fig. 7*A*). Consistent with this, disabling half-(in)activation shifts altogether produced very similar responses to perturbations, with maximal conductances evolving along nearly identical trajectories (Fig. 7*B*). There was one exception in which the slow half-(in)activation mechanism was actively engaged: in this simulation, the first perturbation drove noticeable changes in half-(in)activation parameters, yet the neuron failed to reestablish stable bursting during that perturbation. Disabling half-(in)activation shifts in this case did not restore bursting. Of the remaining 19 models, 16 behaved qualitatively similarly to the representative example shown in Fig. 7.

## Discussion

Some neurons exhibit an intriguing property: they recover more rapidly from repeated perturbations even though their baseline activity appears unchanged, as illustrated by the example model in Fig. 1. At first glance, this behavior is unexpected under a simple homeostatic framework. In such a picture, once a perturbation ends, a neuron relaxes back to its original electrical state; upon re-exposure, recovery would proceed in essentially the same way, with no indication of prior experience. The observation that recovery improves with repeated exposure therefore implies that information about past perturbations is being stored in the system. Computational experiments in Figs. 4 and 7 indicate this information might be stored through persistent adaptations in a neuron’s channel densities. Rapid shifts in channel half-(in)activation then allow the neuron to exploit this modified channel repertoire to recover quickly during subsequent perturbations while still returning to baseline activity during wash, as shown across the population analyses in Figs. 3 and 6. The necessity of coordinating processes across distinct timescales is consistent with observations in synaptic homeostatic plasticity, where multiple pathways operating on different timescales are thought to interact to stabilize synaptic transmission (31, 32).

Crucially, this conclusion would be difficult to demonstrate experimentally. Measuring the full intrinsic state of a neuron would require isolating individual ionic currents sequentially, making it impossible to access the complete channel repertoire at a single moment in time. In contrast, the model allows simultaneous access to all the neuron’s maximal conductances and half-(in)activations, making it possible to track how the intrinsic state evolves over time. This comprehensive view is essential for identifying where the adaptive trace is stored. Likewise, the manipulations performed here—reinitializing neurons with specific combinations of conductances and voltage-dependence (Fig. 4), or selectively altering the timescales governing all intrinsic parameters (Fig. 7)—are not experimentally feasible. Together, these advantages made computational modeling well suited to identify the mechanisms underlying persistent adaptation.

The generality of persistent adaptation across neuronal classes remains unknown. Our experimental motivation comes from two systems in which history-dependent improvement in recovery has been reported: pyloric neurons of the stomatogastric ganglion (STG) exposed to repeated high-potassium perturbations, and RCH in insects. In both cases, available evidence supports improved recovery following prior exposure, but does not establish how widespread this behavior is across neuron types. In the STG, detailed intracellular studies have focused primarily on the pyloric dilator neuron (1, 2, 6), while other identified neurons have not been systematically evaluated for persistent adaptation. Similarly, in studies of RCH in *Locusta migratoria*, evidence for altered neural tolerance derives largely from extracellular recordings of central pattern-generating circuits rather than from cell-type-specific intracellular measurements (5). Thus, while these systems motivate the phenomenon, they do not yet provide a comprehensive map of which neurons do or do not exhibit persistent adaptation. Importantly, such heterogeneity—if present—could itself be functionally meaningful, reflecting differences in the constraints under which neurons with distinct functional roles operate.

The results rest on several conceptual assumptions about how homeostatic regulation might occur. First, the model assumes that homeostasis acts to maintain a fixed activity set point, rather than a broader acceptable range. In biological neurons, a range-based regulation scheme could allow activity to drift within limits, potentially altering the character of the adaptations observed here. Second, the modeled neurons are effectively “naïve,” with no history of prior perturbations before those simulated. Neurons that have already adapted to similar challenges may start closer to an adapted state, limiting the apparent persistent effect. Third, the 20 model neurons studied represent a relatively homogeneous subset of the broader degenerate solution space (*Methods*). While this controlled selection isolates a clean example of persistent adaptation via timescale separation, it does not imply that all parameter combinations behave similarly. Instead, the findings illustrate one plausible mechanism among many that could account for improved recovery. Finally, we assume a simplified representation of excitability where all the firing dynamics are determined by sets of voltage- and time-dependent ion channel processes. Actually, in biological neurons, excitability arises from a richer interplay of mechanisms—for instance specialized channels that support rapid firing (33) or metabolic feedbacks that couple activity to cellular energy use (34). Exploring these assumptions in future work—such as varying the homeostatic rule to target a range or initializing from partially adapted states—could clarify the generality of the observed dynamics. Additional technical assumptions associated with the model are evaluated in Methods.

Persistent, yet often cryptic, intrinsic changes can have both advantageous and maladaptive consequences. Because multiple ion channel configurations can satisfy the same activity target (8, 10, 13, 14, 30), adaptations in channel density may, in principle, place a neuron in a state that is beneficial in one context but maladaptive in another. For example, in neuropathic pain, persistent changes in channel densities can mean that even after the acute injury heals, the neurons remain persistently overly responsive, underpinning chronic pain sensations (35, 36). On the other hand, cryptic variability can be potentially advantageous: in the model, once persistent ion channel adaptation had been established, the fast mechanism allowed the neuron to move rapidly among multiple target-satisfying solutions, enabling quicker recovery from repeated perturbations. The qualifier “potentially” is important because an ion channel density state that enhances recovery from one perturbation may, at the same time, increase vulnerability to a different perturbation. These cryptic changes at the individual-cell level may then propagate to the circuit scale, where intrinsic tuning across neurons collectively shapes network excitability and function (37–40, 41). The computational consequences of these cryptic changes would be interesting to evaluate further (42, 43).

Finally, we note that the persistent adaptation in the maximal conductances “prime” the neuron, so that during subsequent perturbations, fast half-(in)activation shifts immediately to fine‘tune intrinsic currents to recover the target activity. In this way, the neuron’s exposure alters the rules for its future plasticity—embodying plasticity of plasticity, or metaplasticity (44). Further work has shown how this might be implemented biophysically through, for instance, synaptic scaling (45) or changes in postsynaptic receptor subunit composition (46). Our results suggest that intrinsic homeostatic plasticity may play a parallel role, providing a biophysical mechanism for metaplasticity through changes in intrinsic excitability, mediated through alterations in channel density and voltage-dependence.

## Methods

The homeostatic model consisted of a conductance-based neuron, a set of equations that sensed fluctuations in intracellular calcium concentration, and a mechanism that adjusted maximal conductances and channel half-(in)activation voltages. The timescale of maximal conductance adjustments is denoted $\tau_{g}$ and assumed to be 600 s unless noted otherwise. The timescale of half-(in)activation adjustments is denoted $\tau_{\mathrm{half}}$ and is assumed to be 6 s unless noted otherwise. The homeostatic model allowed maximal conductances to vary continuously over positive values and allowed half-(in)activation voltages to shift positively or negatively relative to their baseline values in response to deviations from prespecified target calcium levels. Extreme adjustments are discouraged by the model, but no hard bounds are set. The target calcium levels served as proxies for the neuron’s target activity pattern.

All parameters—including reversal potentials, gating variable time constants, (in)activation curves, calcium sensor parameters, target calcium levels, and the parameters governing how maximal conductances and half-(in)activations are modulated, as well as the integrator’s parameters are described in detail in Mondal et al. (8). The baseline half-activation and half-inactivation values of all the currents used in this work are reproduced in Table 1. The figures in the present study display only shifts in half-(in)activation relative to these values.

**Table 1.** Baseline half-activation and half-inactivation voltages for all ionic currents used in this study

Current	$V_{m_{1/2}}$ (mV)	$V_{h_{1/2}}$ (mV)
I_Na_	–25.5	–48.9
I_CaT_	–27.1	–32.1
I_CaS_	–33	–60
I_H_	–70	
I_Kd_	–12.3	
I_KCa_	–28.3	
I_A_	–27.2	–56.9

All reported shifts in activation and inactivation curves are measured relative to these baseline values. Across the 20 simulations analyzed in Figs. 2 and 3, the largest shift produced by the homeostatic mechanism was less than 6.2 mV in either direction for any current’s activation or inactivation curve.

The 20 models analyzed in the present study are the same 20 models investigated in Mondal et al. (8). Each model begins a simulation with different initial offsets from baseline reference (Table 1), resulting in distinct channel voltage-dependence profiles across the population. All models had a burst period within 20% of the average. The homeostatic mechanism then further shifts these parameters in response to deviations from the target calcium levels.

Perturbations were administered as in Mondal et al. (8). Briefly, increasing the extracellular potassium concentration altered both the potassium reversal potential and the leak reversal potential. Using the Nernst equation, the potassium reversal potential was shifted from –80 mV to –55 mV. Using the method described in Mondal et al. (8), the leak reversal potential was shifted from –50 mV to –32 mV.

### Burst Onset Measurements.

The latency from perturbation onset to the start of regular bursting was measured using a custom Python function that leverages the Electrophys Feature Extraction Library (eFEL) distributed with BluePyOpt (47). For each perturbation trial, the voltage trace and corresponding time vector were formatted as an eFEL trace object and passed to the get_feature_values function together with one of eFEL’s predefined extractable features—peak_time in this case—using eFEL’s default spike detection parameters. This returned the times of all detected spike peaks. Interspike intervals (ISIs) were computed, and the onset of regular bursting was defined as the first spike occurring after the last prolonged quiescent interval (ISI > 5 s). If no ISIs exceeded this threshold, the time of the first detected spike was taken as the burst onset. This procedure was applied to each perturbation trial of each neuron to produce the latency values plotted in Fig. 2*A*.

### Quantifying Parameter Space Trajectories.

To quantify changes in intrinsic properties over time, we extracted each neuron’s full set of maximal conductances [or half-(in)activation voltages] at seven predefined simulation time points spanning perturbation and wash phases: just before each of perturbations 1, 2, and 3; at the end of each perturbation; and after the final wash (seven points total). Values were averaged over a 20 s window (from 30 s to 10 s before the event) to avoid selecting a single time point arbitrarily. The neuron’s parameters were already stable over this period, so the averaging does not affect the measured values.

For each neuron, trajectories in conductance space and half-(in)activation space were characterized by computing vectors between successive points, obtained by subtracting one point from the next. The Euclidean distance of each vector was calculated by squaring each element, summing across dimensions, and taking the square root. This yielded six vector lengths per neuron for maximal conductances, and another six for half-(in)activations.

To characterize the adapted conductance regime, we analyzed the six states following the first perturbation (points 2 to 7). Their mean was taken as the center of the adapted region in conductance space. The Euclidean distance from each point to this center was then computed, and the multidimensional sphere radius was defined as the largest of these distances—representing the smallest sphere centered at the mean that enclosed all postadaptation states. We then measured the Euclidean distance from the conductance state prior to the first perturbation to the center of the adapted region. Subtracting the sphere radius from this distance yielded how far outside the final adapted regime the initial state lay.

To characterize the behavior in half-(in)activation space, we examined whether the trajectory in half-(in)activation space reversed direction between repeated visits to the same condition. We focused on two sequences late in the simulation: the final wash–perturbation–wash cycle, and the final perturbation–wash–perturbation cycle. In each case, we treated the two consecutive transitions as vectors in parameter space and calculated the angle between them using the inverse of the dot-product relationship between vector direction and angle. Angles were reported in degrees. Values greater than 90 degrees indicated that the trajectory had reversed direction between visits to the same regime.

### Evaluating Technical Assumptions.

Our homeostatic model makes several simplifying assumptions. First, all channels within a given regulatory mode share the same time constant, and posttranslational modifications are removed at the same rate as they are added. In reality, individual channels likely differ in their modification and turnover kinetics, and addition/removal rates may be asymmetric. In fact, the rates of their modification may depend on how the ion channels colocalize with each other (48). However, as long as posttranslational changes remain collectively much faster than expression-level changes, the separation of timescales that drives our results should hold. Second, posttranslational regulation is implemented solely as midpoint shifts in (in)activation curves with fixed slope, and these shifts are bounded by uniform limits across channels. While biological changes can also alter slope, and the permissible range may differ between channels, these details would alter the magnitude but not the existence of the fast–slow division of labor we observe. Third, we assume that all activity-dependent changes are driven directly by calcium influx, with both regulatory modes sharing the same target activity. In practice, calcium signals can engage distinct downstream pathways, and additional coregulation or neuromodulatory effects may introduce separate set points for different channels. Such extensions could change the quantitative trajectory of adaptation but would not remove the qualitative role of the faster process in rapid tuning and the slower process in storing persistent adaptations, provided that their characteristic timescales remain well separated.

## Data Availability

Zip file data have been deposited in Zenodo https://doi.org/10.5281/zenodo.18188912 (49).
